# Serum angiopoietin-2/angiopoietin-1 ratio is associated with cardiovascular and all-cause mortality in peritoneal dialysis patients: a prospective cohort study

**DOI:** 10.1080/0886022X.2024.2380037

**Published:** 2024-07-31

**Authors:** Han Li, Qianhui Song, Xinyu Su, Yiwei Shen, Hao Yan, Zanzhe Yu, Zhenyuan Li, Jiangzi Yuan, Jiaying Huang, Zhaohui Ni, Leyi Gu, Wei Fang

**Affiliations:** a Department of Nephrology, Renji Hospital, School of Medicine, Shanghai Jiao Tong University, Shanghai, China; b Shanghai Center for Peritoneal Dialysis Research, Shanghai, China

**Keywords:** Angiopoietin-2, angiopoietin-1, peritoneal dialysis, cardiovascular mortality, all-cause mortality

## Abstract

**Introduction:**

Our objective was to examine the factors associated with the serum angiopoietin-2/angiopoietin-1 (Angpt-2/Angpt-1) ratio in peritoneal dialysis (PD) patients and to investigate the association between Angpt-2/Angpt-1 ratio and cardiovascular and all-cause mortality.

**Methods:**

Patients on PD who were prevalent between January 2014 and April 2015 in the center of Renji Hospital were enrolled. At the time of enrollment, serum and dialysate samples were collected to detect biochemical parameters, serum angiopoietin-2 and angiopoietin-1 levels. Patients were dichotomized into two groups according to a median of Angpt-2/Angpt-1 ratio and followed up prospectively until the end of the study.

**Results:**

A total of 325 patients were enrolled, including 168 males (51.7%) with a mean age of 56.9 ± 14.2 years and a median PD duration of 32.4 (9.8-55.9) months. Multiple linear regression showed pulse pressure (β = 0.206, *p* < .001) and high-sensitivity C-reactive protein (hs-CRP) (β = 0.149, *p* = .011) were positively correlated with serum Angpt-2/Angpt-1 ratio, while residual renal function (RRF) (β= −0.219, *p* < .001) was negatively correlated with serum Angpt-2/Angpt-1 ratio. Multivariate Cox regression analysis showed the high serum Angpt-2/Angpt-1 ratio was an independent predictor of cardiovascular mortality (hazard ratio (HR)=2.467, 95% confidence interval (CI) 1.243–4.895, *p* = .010) and all-cause mortality (HR = 1.486, 95%CI 1.038–2.127, *p* = .031). In further subgroup analysis by gender, a significant association was shown in high Angpt-2/Angpt-1 ratio with all-cause mortality in male (*p* < .05), but not in female patients (*p*>.05).

**Conclusions:**

High Angpt-2/Angpt-1 ratio is an independent risk factor for cardiovascular and all-cause mortality in PD patients.

## Introduction

Peritoneal dialysis (PD) is a well-established renal replacement therapy for patients with end-stage renal disease (ESRD). Cardiovascular disease (CVD) is the leading cause of mortality in patients on PD, and approximately 40%–60% of deaths in the PD population are due to cardiovascular events [1], which are caused by both traditional and nontraditional factors. However, the exact mechanism underlying the high CVD mortality is still not fully understood. Angiopoietins (Angpts) are a group of vascular growth factors that play critical roles in vascular remodeling, maturation, and stabilization [2–5]. The Angpts family includes Angpt-1, Angpt-2, Angpt-3, and Angpt-4, which interact with receptors tyrosine kinase Tie [2]. Angpt-1 and Angpt-2 have antagonistic effects on Tie2. Angpt-1 induces phosphorylation of Tie2 and promotes endothelial cell migration and survival to maintain vascular stabilization [6, 7]. In contrast, Angpt-2 can compete with Angpt-1 for binding to Tie2 to downregulate Tie2 phosphorylation, thereby disrupting vascular stabilization and sensitizing endothelial cells to pro-inflammatory cytokines and other endothelial growth factors, resulting in vascular endothelial activation and inflammation [8]. The Angpt-2/Angpt-1 ratio has been reported to be closely related to the development risk and outcome of various diseases [9–18]. Our previous study demonstrated that high Angpt-2 levels is an independent predictive factor for fatal and non-fatal cardiovascular events in PD patients [19]. However, whether Angpt-2/Angpt-1 ratio is associated with the outcomes of patients with PD remains unclear. Therefore, we conducted a prospective cohort study to examine the factors associated with the serum Angpt-2/Angpt-1 ratio in PD patients and investigated the association of the Angpt-2/Angpt-1 ratio with cardiovascular mortality and all-cause mortality in PD patients.

## Materials and methods

### Study participants

Stable patients who received continuous ambulatory peritoneal dialysis (CAPD) in the PD center of Renji Hospital, School of Medicine, Shanghai Jiao Tong University, between January 2014 and April 2015 were enrolled. Exclusion criteria included: (1) acute cardiocerebrovascular events that occurred within 3 months prior to the study; (2) the presence of systemic inflammatory disease, including chronic autoimmune disorders, peritonitis, exit-site infection, or other acute infective complications in the preceding 3 months; (3) malignancy; (4) chronic liver disease, chronic rheumatic heart disease, and congenital heart disease; (5) use of glucocorticoids or immunosuppressive agents during the past 1 year; and (6) refusal to provide written consent or incomplete data. All the patients were dialyzed using lactate-buffered glucose-based PD solutions (Dianeal^®^, Baxter, China). This study was approved by the Human Research Ethics Committee of Renji Hospital, School of Medicine, Shanghai Jiao Tong University, and all participants provided written informed consent.

### Data collection

The following demographic data were collected at the time of enrollment: age, gender, PD duration, underlying disease of ESRD, presence of comorbidity, and whether taking angiotensin-converting enzyme inhibitors/angiotensin II receptor blockers (ACEI/ARB). The comorbidities included diabetes, hypertension, and CVD. CVD was defined as the presence of ischemic heart disease, history of angina, previous myocardial infarction, coronary artery bypass surgery or stenting, New York Heart Association functional class III to IV congestive heart failure, cerebrovascular events, transient ischemic attack, or peripheral arterial disease with or without amputation.

At the time of enrollment, fasting venous blood samples were collected from each patient to measure serum Angpt-1 and Angpt-2 levels. Serum Angpt1 and Angpt-2 levels were determined using enzyme-linked immunosorbent assay (ELISA) kits (R&D Systems Inc., Minneapolis, MN, USA). All samples were run simultaneously and in duplicate to avoid intra- and inter-assay variation. Serum albumin, high-sensitivity C-reactive protein (hs-CRP), and hemoglobin levels were also measured.

Patients’ dialysis prescriptions were collected, and glucose exposure was calculated. Small solute clearance was determined by measuring total weekly urea clearance (Kt/V) and creatinine clearance (CrCl) using standard methods. The residual renal function (RRF) was calculated as the average 24-h urine urea and creatinine clearance. At enrollment, a standard peritoneal equilibration test (PET) was performed for each patient.

### Follow-up

All patients were prospectively followed from enrollment until death, transfer to permanent hemodialysis, kidney transplantation, transfer to other centers, PD cessation due to renal function recovery, or the end of the study (June 30, 2022). Patients were stratified into a low Angpt-2/Angpt-1 ratio group and a high Angpt-2/Angpt-1 ratio group according to the median value of Angpt-2/Angpt-1 ratio, and the outcome of the patients was recorded. The primary endpoints were cardiovascular mortality and all-cause mortality. Cardiovascular mortality was defined as death caused by cardiovascular disease, including fatal coronary heart disease, sudden cardiac mortality, and fatal heart failure. All-cause mortality was defined as the total death due to any cause.

### Statistical analysis

Continuous data were expressed as mean ± SD or median (interquartile range), depending on the distribution of data. Count data were expressed as frequencies (%). Unpaired *t*-tests or Mann–Whitney tests were used to compare different groups depending on whether the data were normally skewed or distributed. Categorical variables were compared using the chi-squared test. Multiple linear regression analysis was performed to assess the independent factors associated with Angpt-2/Angpt-1 ratio. Cumulative survival curves were generated using the Kaplan–Meier method and compared using the log-rank test. The Cox proportional hazard regression model was used to estimate the relative risks of cardiovascular and all-cause mortality. Important demographic characteristics, biochemical parameters and factors with *p* < .2 in the univariate Cox analysis for cardiovascular mortality and all-cause mortality were further entered into the multivariate Cox regression analysis. Statistical significance was set at *p* < .05. Statistical analysis was performed using the SPSS software (IBM Corp. Released 2019. IBM SPSS Statistics for Windows, Version 26.0. Armonk, NY: IBM Corp).

## Results

### Patients’ characteristics

A total of 325 patients (168 male) were enrolled in this study, with a mean age of 56.9 ± 14.2 years and a median PD duration of 32.4 (9.8–55.9) months. Among them, 82 (25.2%) were comorbid with diabetes, 298 (91.7%) had hypertension, and 127 (39.1%) had a history of CVD. The primary kidney diseases were chronic glomerulonephritis in 105 patients (32.3%), diabetic nephropathy in 46 patients (14.2%), hypertensive nephrosclerosis in 14 patients (4.3%), polycystic kidney disease in 10 patients (3.1%), obstructive nephropathy in 6 patients (1.8%), others and the renal diagnosis was unknown in 149 patients (45.8%) (shown in Table 1).

**Table 1.** Characteristics of the study population (*n* = 325).

Variables	All PD patients (*n* = 325)	Low Angpt-2/Angpt-1 ratio group (*n* = 163)	High Angpt-2/Angpt-1 ratio group (*n* = 162)	*p*-value
Gender (Male)	168 (51.7%)	83 (50.9%)	85 (52.5%)	.780
Age (years)	56.9 ± 14.2	55.7 ± 14.3	58.1 ± 14.1	.126
Pulse pressure(mmHg)	53 ± 15	53 ± 15	53 ± 16	.753
PD duration (months)	32.4 (9.8–55.9)	26.0 (6.7–50.0)	35.2 (14.2–62.1)	.019
Underlying renal disease [*n* (%)]				
Chronic glomerulonephritis	105 (32.3%)	58 (35.6%)	47 (29.0%)	
Diabetic nephropathy	46 (14.2%)	22 (13.5%)	24 (14.8%)	
Hypertension	14 (4.3%)	8 (4.9%)	6 (3.7%)	
Polycystic kidney disease	10 (3.1%)	5 (3.1%)	5 (3.1%)	
Obstructive nephropathy	6 (1.8%)	0 (0)	6 (3.7%)	
Others and Unknown	149 (45.8%)	72 (44.2%)	78 (47.5%)	
Comorbidity [*n* (%)]				
Diabetes mellitus	82 (25.2%)	41 (25.2%)	41 (25.3%)	.974
Hypertension	298 (91.7%)	145 (89.0%)	153 (94.4%)	.073
Cardiovascular disease	127 (39.1%)	60 (36.8%)	67 (41.4%)	.401
ACEI/ARB taking [*n* (%)]	191 (58.8%)	100 (61.3%)	91 (56.2%)	.343
Previous peritonitis episode [*n* (%)]	79 (24.3%)	41 (25.2%)	38 (23.5%)	.698
Historical glucose exposure (g/year)	43,800 (32,850–55,642)	40,150 (32,850–49,434)	49,119 (39,707–58,400)	<.001
Hemoglobin (g/L)	107.7 ± 17.0	107.6 ± 16.6	107.8 ± 17.4	.926
Serum albumin (g/L)	37.0 ± 4.6	37.0 ± 4.7	36.9 ± 4.6	.761
LDL cholesterol (mmol/L)	2.68 (2.04–3.28)	2.59 (1.92–3.26)	2.72 (2.19–3.36)	.282
Hs-CRP (mg/L)	2.55 (0.90–6.59)	1.55 (0.60–5.20)	3.44 (1.38–7.70)	<.001
Total Kt/V urea	1.93 ± 0.36	1.98 ± 0.41	1.88 ± 0.31	<.001
Total CrCl (L/week/1.73 m^2^)	62.31 ± 18.32	66.47 ± 20.17	58.13 ± 15.20	<.001
RRF (ml/min)	0.96 (0–2.89)	1.91 (0.34–3.83)	0.35 (0–1.90)	<.001
Urine output (ml/24 h)	300 (0–800)	520 (175–1100)	150 (0–500)	<.001
UF (ml/24 h)	460 (18–828)	315 (−85–650)	637 (310–925)	<.001

Values expressed as mean ± standard deviation, median (25–75th percentile), or absolute numbers with percentages [*n* (%)]. Angpt-2/Angpt-1: angiopoietin-2/angiopoietin-1; PD: peritoneal dialysis; ACEI/ARB: inhibitor/ angiotensin receptor blocker; LDL: lower-density lipoprotein; Hs-CRP: high sensitivity C-reactive protein; Kt/V urea: urea clearance index; CrCl: creatinine clearance; RRF: residual renal function; UF: ultrafiltration.

### Factors associated with Angpt-2/Angpt-1 ratio

In this cohort, the level of Angpt-1 was 42015.0 (28188.5–63361.3) pg/ml, and the level of Angpt-2 was 5438.3 (3397.6–7691.0) pg/ml. The median serum Angpt-2/Angpt-1 ratio in the present cohort was 0.133. Patients were dichotomized into low (Angpt-2/Angpt-1 ratio ≤ 0.133) and high (Angpt-2/Angpt-1 ratio > 0.133) groups by the median. Compared with patients in the low Angpt-2/Angpt-1 ratio group, patients in the high Angpt-2/Angpt-1 ratio were more likely to have longer PD duration (*p* = .019), higher total glucose exposure (*p* < .001), hs-CRP (*p* < .001) and 24-h ultrafiltration volume (*p* < .001), lower total Kt/V (*p* < .001), total CrCl (*p* < .001), 24-h urine volume (*p* < .001) and RRF (*p* < .001). No significant differences were observed in other demographic characteristics, laboratory data, and indicators for dialysis between patients in the low Angpt-2/Angpt-1 ratio group and the high Angpt-2/Angpt-1 ratio group (shown in Table 1).

As shown in Table 2, after adjusting for age, gender, glucose exposure, comorbid with CVD, DM, hemoglobin and serum albumin, pulse pressure (β = 0.206, *p* < .001) and high-sensitivity C-reactive protein (hs-CRP) (β = 0.149, *p* = .011) were positively correlated with serum Angpt-2/Angpt-1 ratio, while RRF (β = −0.219, *p* < .001) was negatively correlated with serum Angpt-2/Angpt-1 ratio.

**Table 2.** Multiple linear regression analysis of Angpt-2/Angpt-1 ratio and patient characteristics.

Variables	β	*p*-value
Gender (Male)	−0.035	.524
Age (years)	−0.059	.338
Pulse pressure (mmHg)	0.206	<.001
Historical glucose exposure (g/year)	−0.025	.641
Cardiovascular disease	0.070	.233
Diabetes mellitus	−0.032	.591
Hemoglobin (g/L)	−0.013	.817
Serum albumin (g/L)	−0.045	.412
Log_10_hs-CRP	0.149	.011
RRF (ml/min)	−0.219	<.001

Angpt-2/Angpt-1: angiopoietin-2/angiopoietin-1; Log_10_ hs-CRP: the logarithm of high sensitivity C-reactive protein to the base 10; RRF: residual renal function.

### Serum Angpt-2/Angpt-1 ratio and outcome of patients

After being followed up for 47.4 (24.4–82.3) months, 132 patients died, 77 were transferred to permanent hemodialysis, 34 received kidney transplantation, 14 were transferred to other centers, and 2 were lost to follow-up. The causes of death included CVD (*n* = 40), cerebrovascular events (*n* = 23), infections (*n* = 37), malignancies (*n* = 7), malnutrition (*n* = 2), withdrawal of treatment (*n* = 4), other causes (*n* = 9), and unknown causes (*n* = 10).

**Figure 1.**
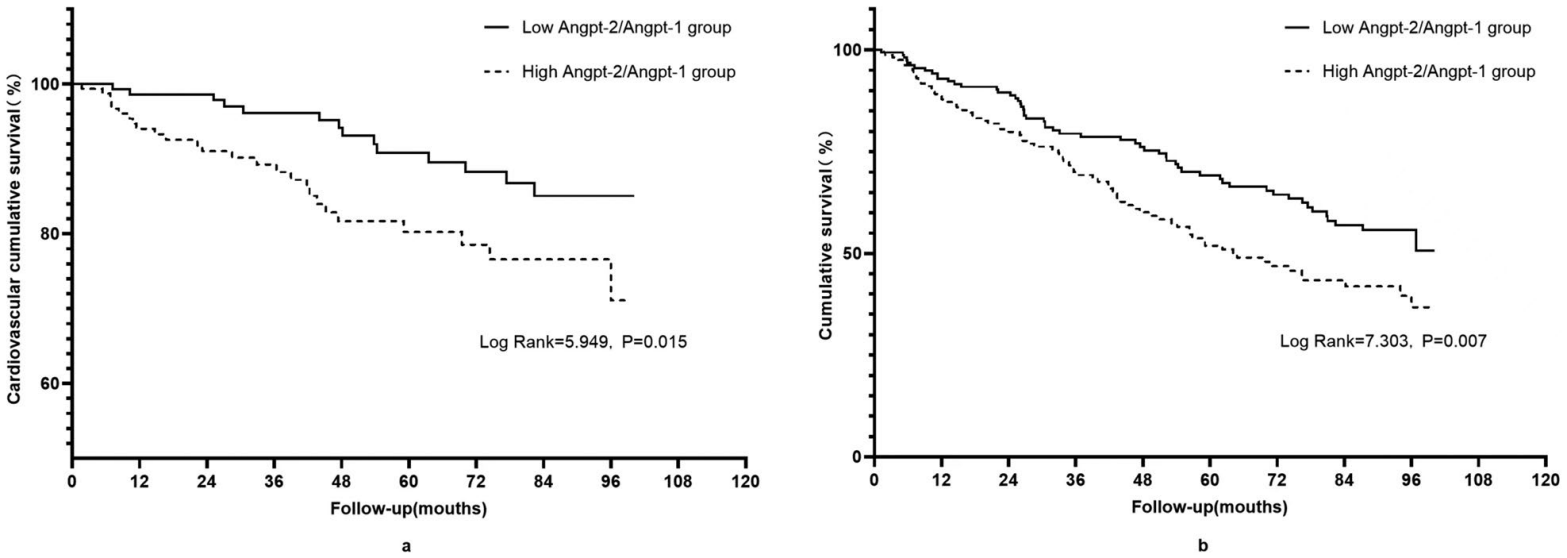
Kaplan–Meier estimates of cardiovascular mortality (a) and all-cause mortality (b) survival probability of patients stratified by the median of serum Angpt-2/Angpt-1 ratio. Lower group: Angpt-2/Angpt-1 ratio ≤ 0.133, high group: Angpt-2/Angpt-1 ratio > 0.133.

As shown in Figure 1, compared with the patients in the low Angpt-2/Angpt-1 ratio group, a significant increase in cardiovascular mortality (log-rank = 5.949, *p* = .015) and all-cause mortality (log-rank = 7.303, *p* = .007) were observed in patients in the high Angpt-2/Angpt-1 ratio group (shown in Figure 1). The univariate Cox analysis was used to examine factors in relation to cardiovascular and all-cause mortality (online Supplementary Table 2). Multivariate Cox regression analysis showed serum Angpt-2/Angpt-1 ratio was an independent predictor of cardiovascular mortality (HR = 2.467, 95%CI 1.243–4.895, *p* = .010; shown in Table 3) and all-cause mortality (HR = 1.486, 95%CI 1.038–2.127, *p* = .031; shown in Table 4) after adjusting for gender, age, comorbid with CVD, diabetes, hemoglobin, serum albumin, hs-CRP, and residual renal function.

**Table 3.** Multivariate Cox regression models for cardiovascular mortality.

Variables	HR (95% CI)	*p*-value
Gender (Male)	1.056 (0.537–2.08)	.874
Age (years)	1.052 (1.019–1.086)	.002
Cardiovascular disease	6.360 (2.85–14.189)	<.001
Diabetes mellitus	1.850 (0.918–3.727)	.085
Hemoglobin (g/L)	0.989 (0.971–1.008)	.248
Serum albumin (g/L)	0.975 (0.906–1.050)	.500
Log_10_hs-CRP	1.104 (0.556–2.191)	.778
RRF (ml/min)	1.043 (0.890–1.222)	.604
Angpt-2/Angpt-1 ratio	2.467 (1.243–4.895)	.010

HR: hazard ratio; 95%CI: 95% confidence interval; Log_10_hs-CRP: the logarithm of high sensitivity C-reactive protein to the base 10; RRF: residual renal function; Angpt-2/Angpt-1: angiopoietin-2/angiopoietin-1.

**Table 4.** Multivariate Cox regression models for all-cause mortality.

Variables	HR (95% CI)	*p*-value
Gender (Male)	0.980(0.689–1.395)	.912
Age (years)	1.050(1.032–1.067)	<.001
Cardiovascular disease	2.959(2.000–4.378)	<.001
Diabetes mellitus	1.342(0.902–1.995)	.147
Hemoglobin (g/L)	0.995(0.985–1.005)	.352
Serum albumin (g/L)	0.983(0.943–1.024)	.403
Log_10_hs-CRP	1.217(0.860–1.721)	.267
RRF (ml/min)	0.925(0.842–1.016)	.104
Angpt-2/Angpt-1 ratio	1.486(1.038–2.127)	.031

HR: hazard ratio; 95%CI: 95% confidence interval; Log_10_hs-CRP: the logarithm of high sensitivity C-reactive protein to the base 10; RRF: residual renal function; Angpt-2/Angpt-1: angiopoietin-2/angiopoietin-1.

**Figure 2.**
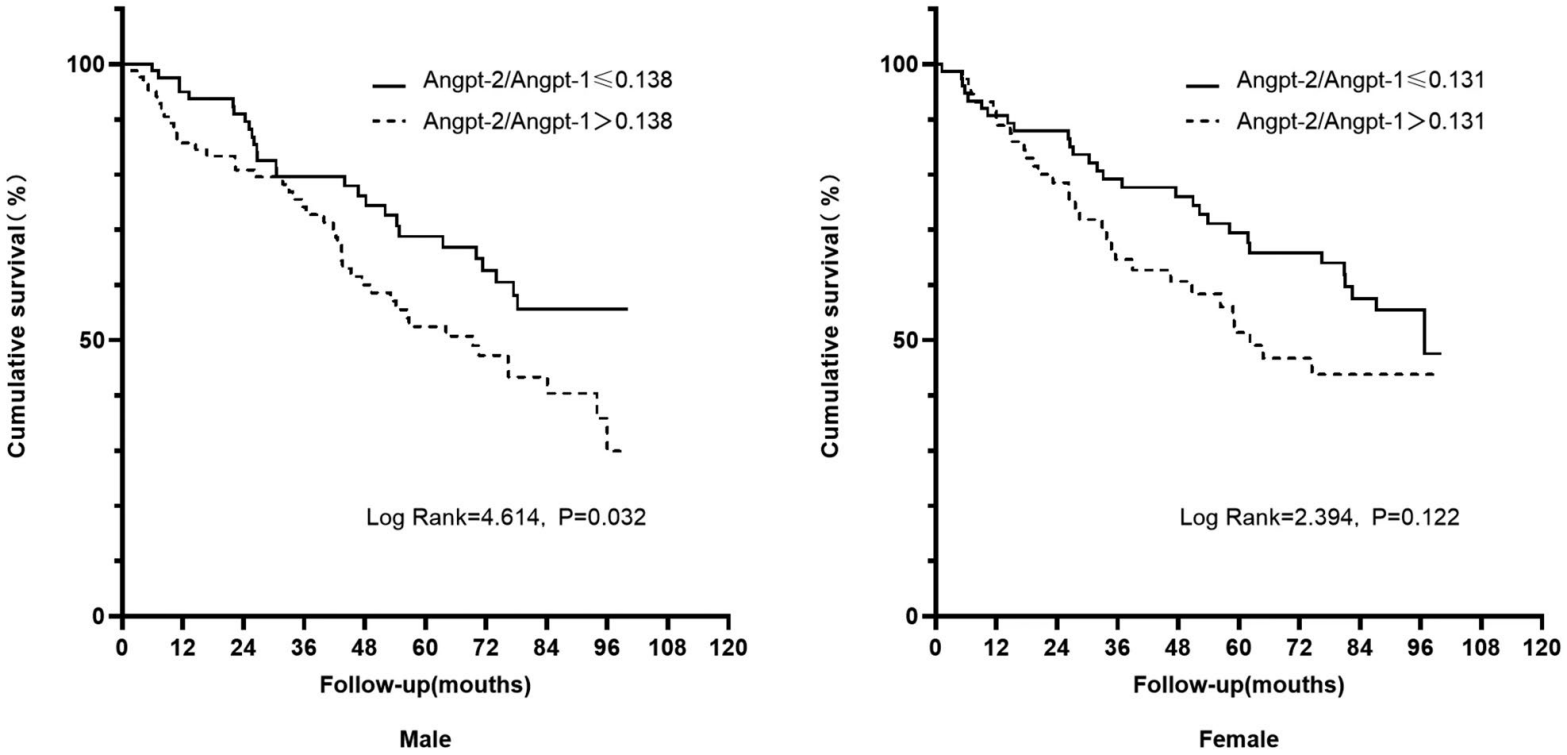
Kaplan–Meier estimates of all-cause mortality. Patients were divided according to median of Angpt-2/Angpt-1 ratio in male or female study participants.

In the subgroup analysis by gender, a significant association was shown in high Angpt-2/Angpt-1 ratio with all-cause mortality in male (log-rank = 4.614, *p* = .032, shown in Figure 2), but not in female patients (log-rank = 2.394, *p* = .122, shown in Figure 2). We further performed multivariable Cox regression analysis, results were shown a significant predictive effect of Angpt-2/Angpt-1 ratio on all-cause mortality in the male participants as well as in the whole cohort, but not in the female participants (shown in Supplementary Table 3). We did not conduct a subgroup analysis for cardiovascular mortality due to the small number of events.

## Discussion

In the present study, we reported an association between the serum Angpt-2/Angpt-1 ratio and cardiovascular mortality and all-cause mortality in patients with PD. Pulse pressure (PP), hs-CRP, and RRF were independently associated with Angpt-2/Angpt-1 ratio in this cohort. An increased Angpt-2/Angpt-1 ratio is an independent risk factor for cardiovascular mortality and all-cause mortality in PD patients.

We found that PP was independently associated with Angpt-2/Angpt-1 ratio in patients with PD. Pulse pressure is an essential indicator to reflect the degree of arterial stiffness [20]. A prospective cohort study of 416 patients with chronic kidney disease (CKD) in Taiwan also showed that plasma Angpt-2 levels was independently associated with arterial stiffness severity as measured by pulse wave conduction velocity (PWV) [21]. Studies have found that accelerated arterial stiffness was more pronounced in the chronic hemodialysis patients than in the general population patients [22] and PWV was a strong independent predictor of overall and cardiovascular mortality in patients undergoing dialysis [23]. It has been reported that PP is positively associated with circulating Angpt-2 [24] and Tie-2 [25]. In addition, experimental data have suggested that Angpt-1 favorably influences vascular remodeling through anti-inflammatory and anti-atherosclerosis properties [26,27], whereas Angpt-2 competes with Angpt-1 for binding to Tie2 [8,28]. Therefore, the deregulation of Angpt-1 and Angpt-2 is closely related to vascular remodeling and arterial stiffness, which may explain the association between PP and Angpt-2/Angpt-1 ratio.

High serum hs-CRP levels in PD patients were also associated with Angpt-2/Angpt-1 ratio in our cohort. Angpt-1 is expressed in smooth muscle cells and pericytes, and the Angpt-1-Tie2 signaling pathway exerts anti-inflammatory properties by increasing pericyte coverage, promoting vascular endothelial cell survival, stabilizing blood vessels with intercellular connections [29–31], and inhibiting the NF-κB pathway [32]. Angpt-2 is produced in endothelial cells and stored in Weibel-Palade bodies (WPBs) within endothelial cells [33]. It has been reported that pro-inflammatory factors such as TNF-α [34] and IL-6 [35] can act on WPBs to increase the release of Angpt-2 by endothelial cells. Angpt-2 inhibits Angpt-1-mediated phosphorylation of Tie2. Moreover, Angpt-2 locally regulates pericyte migration and coverage [36]. Elevated serum Angpt-2 and lower serum Angpt-1 levels have been reported in mouse models of pneumococcal pneumonia [37] and patients with inflammatory bowel disease (IBD) [38–40]. Thus, the deregulation of Angpt-2 and Angpt-1 might be closely associated with inflammation, and hs-CRP is a recognized marker of systemic inflammation, which can account for the close correlation between the Angpt-2/Angpt-1 ratio and hs-CRP.

Our study showed that RRF correlates inversely with the serum Angpt-2/Angpt-1 ratio in patients with PD. One of the causes of chronic inflammation in patients with PD is the accumulation of uremic toxins and reduced clearance of inflammatory factors due to renal dysfunction. Several uremic toxins can cause oxidative stress, cell adhesion, and inflammatory responses, resulting in endothelial dysfunction [41–43]. High levels of asymmetric dimethylarginine in circulation can reduce nitric oxide production through inhibition of nitric oxide synthase, leading to vascular endothelial dysfunction and excessive secretion of Angpt2 [44,45]. A reduction in elevated Angpt2 was observed after kidney transplantation [46], which may be explained by the increased toxin clearance function after kidney transplantation. Previous studies have found that RRF in PD patients is closely correlated with microinflammation [47], and the residual kidney is the main way to eliminate microinflammatory factors. Compared to healthy individuals, serum Angpt-1 levels decreased and serum Angpt-2 levels increased in non-dialysis and dialysis stage 5 CKD patients [46]. Other studies have found that plasma Angpt2 was positively correlated with CKD staging, but there was no difference between Angpt1 and CKD staging [16]. In the present study, we observed that RRF in patients with PD was inversely correlated with the Angpt-2/Angpt-1 ratio, which may be interpreted as the accumulation of uremic toxins and chronic inflammation.

We found that an increased Angpt-2/Angpt-1 ratio is an independent risk factor for cardiovascular and all-cause mortality in patients with PD. David et al. found that higher levels of Angpt-2 in patients with CKD were independently associated with all-cause mortality [48]. In a prospective cohort study of 313 hemodialysis patients, Chu et al. reported that baseline Angpt-2 was independently associated with all-cause mortality in hemodialysis patients [49]. In a multicenter prospective cohort study of 1503 hospitalized patients, a higher Angpt-1/Angpt-2 ratio was associated with a lower incidence of heart failure and mortality in patients with acute kidney injury [16]. Similarly, our study showed that an increased Angpt-2/Angpt-1 ratio is associated with cardiovascular mortality and all-cause mortality in patients with PD. There are several possible explanations for this observation.

A high Angpt-2/Angpt-1 ratio is a marker of endothelial dysfunction, which is closely associated with atherosclerosis. Experimental data suggested that Angpt-1 favorably influences vascular remodeling through anti-atherosclerosis properties [27]. In vitro studies have shown that Angpt-1 can prevent cardiac arteriosclerosis and myocardial cell apoptosis, thereby improving myocardial cell survival in rats [27]. Decreased Angpt1 has a potential adverse effect on cardiac structural changes [50]. In addition, high Angpt-2 levels in atherosclerotic diseases have been found in nonobstructive coronary artery disease [51], carotid artery disease [52] and peripheral artery disease [53]. Previous studies have found that circulating Angpt-2 is a driver of accelerated athero­sclerosis in patients with CKD, rather than merely a bio­marker of CVD events [44,46]. Inhibition of Angpt-2 improves ischemia-reperfusion injury in the heart, whereas overexpression of Angpt-2 increases cardiac fibrosis and vascular instability in rat and mouse models [54,55]. Studies have found that the circulating Angpt-2 level in patients with type 2 diabetes is increased, but the increase in Angpt1 is not obvious, which results in a significant increase in the Angpt-2/Angpt-1 ratio [56,57]. Therefore, the Angpt-2/Angpt-1 ratio may be a marker of vascular injury in various pathological conditions and is more sensitive than a single measurement of Angpt-1 or Angpt-2. Therefore, patients with PD with a high serum Angpt-2/Angpt-1 ratio may have more severe endothelial dysfunction and vascular changes.

Angpt-2/Angpt-1 ratio is a marker of systemic inflammation. Angpt-1 can inhibit inflammatory processes [4]. Studies have found that Angpt-2 sensitizes endothelial cells to inflammatory cytokines, leading to increased local permeability and recruitment of inflammatory mononuclear/macrophage cells to the vascular wall [34]. In patients with stage CKD3-5 CKD, high Angpt-2 levels were positively correlated with markers of systemic inflammation [44,46,48]. Loss of balance in Angpt-1 and Angpt-2 in patients with pneumonia predicts mortality and length of hospital stay [37]. The deregulation of Angpt-2 and Angpt-1 may lead to endothelial cell activation and chronic inflammation. Quite a few studies have found that Angpt-2/Angpt-1 ratio is closely related to the outcome of patients with severe systemic inflammatory diseases such as sepsis and severe dengue fever [58–60]. The incidence of adverse clinical outcomes in patients with PD is related to chronic inflammation [61–63]. Thus, a high serum Angpt-2/Angpt-1 ratio is associated with an increased risk of mortality owing to inflammation.

In addition to conventional cardiovascular risk factors such as age, sex and comorbidities etc., endothelial dysfunction and chronic inflammation highlight the impact of damage to the vascular wall. Our findings contributed to the current body of literature that the imbalance of the Angpts system plays an important role in endothelial dysfunction and inflammation. In the present study, we found the high Angpt-2/Angpt-1 ratio remained as a potential predictor for cardiovascular and all-cause death in patients on PD after adjusting for multiple risk factors known to be associated with mortality in dialysis population, i.e. age, DM, hypertension, background CVD, serum albumin, hs-CRP and RRF. Based on these findings, larger multicenter studies are warranted as well as preclinical analysis to evaluate the mechanistic roles and cutoff values of Angpt-2/Angpt-1 ratio in the survival of dialysis patients, which could help clinicians identify high-risk patients and allows for targeted monitoring and interventions that may improve their outcomes. Moreover, Angpt-2/Angpt-1 ratio is most likely not just a mortality biomarker, but rather a player in the pathogenesis of vascular damage in PD patients, whether imbalance of Angpts can be arrested by targeted strategies could be interesting to be explore in future studies.

We also found a gender-dependent association between increased Angpt-2/Angpt-1 ratio and all-cause mortality in our study. Male patients in the high Angpt-2/Angpt-1 ratio group showed a significantly inferior survival rate, but this finding was not observed in female patients. After adjustment for patients’ characteristics, multivariable Cox regression analysis showed that Angpt-2/Angpt-1 ratio was an independently predictor of all-cause mortality in male patients on PD, but not in female patients. Similar findings have been reported in a prospective cohort study conducted in 340 HD patients, which showing that Angpt-2 could predict all-cause mortality in male but not female HD patients [49]. Sexual dimorphism in Angpts levels has been reported by several studies. Silha et al. found that significantly higher Angpt-2 levels were observed in females when compared to male overweight and obese subjects [64]. A community-based cohort study of 3778 participants also showed females had higher circulating Angpt-2 and lower soluble Tie2 levels when compared to males [24]. On the other hand, previous experimental and clinical evidence demonstrated that at least a part of cardiovascular benefits of 17 beta-estradiol can be attributed to the direct effect of the ovarian sex steroid hormone on vascular endothelial cells [65]. Several studies have reported a protective effect of female gender in response to traumatic hemorrhagic shock [65–67], which causes a systemic inflammatory response that activates the endothelium. Therefore, we suppose that males may be more sensitive than females to Angpts in patients on PD, which is related to endothelial dysfunction, inflammation and atherosclerosis and thus shows a significant association with all-cause mortality.

Our study has several limitations. First, this was a single-center observational study. Angpts are not routine clinical markers and it has been suggested that serum Angpts levels measured by different laboratories vary widely due to methodological difficulties, so the results of present study need to be verified in multicenter clinical studies by uniform measure methods. Also, serum Angpt-2 and Angpt-1 were only measured once, and the association of longitudinal change with patient outcome needs further study. Finally, although we made substantial efforts to adjust for potential confounding factors, unmeasured confounding factors may still exist. We did not collect the data on smoking status and body mass index and therefore cannot clarify whether this prognostic significance of Angpt-2/Angpt-1 ratio is independent of them.

In summary, pulse pressure, hs-CRP level, and RRF were independently associated with the serum Angpt-2/Angpt-1 ratio. An increased Angpt-2/Angpt-1 ratio is an independent risk factor for cardiovascular and all-cause mortality in patients with PD.

## Supplementary Material

Supplemental Material

Figure 2.jpg

Supplementary table.docx

Figure 1.jpg

## Data Availability

All data generated or analyzed during this study are included in this article. Further inquiries can be directed to the corresponding authors.
