# Supplementary material for: Serum angiopoietin-2/angiopoietin-1 ratio is associated with cardiovascular and all-cause mortality in peritoneal dialysis patients: a prospective cohort study
Source: Ren Fail. 2024 Jul 31;46(2):2380037. doi: 10.1080/0886022X.2024.2380037 (PMC11293270; doi:10.1080/0886022X.2024.2380037)
Supplement: Supplemental Material [file IRNF_A_2380037_SM5378.docx]

**Tables**

**Supplementary Table 1** Spearman correlations of Angpt-2/Angpt-1 ratio and other demographics, biochemical variables, peritoneal transport.

| **Variables** | r | P-value |
| --- | --- | --- |
| **Age** | 0.093 | 0.096 |
| **Pulse pressure(mmHg)** | 0.150 | 0.007 |
| **Historical glucose exposure (g/year)** | 0.279 | ＜0.001 |
| **Hemoglobin (g/L)** | -0.024 | 0.670 |
| **Serum albumin (g/L)** | -0.021 | 0.710 |
| **LDL cholesterol** | 0.027 | 0.627 |
| **Log**10hs-CRP | 0.235 | ＜0.001 |
| **Total Kt/V urea** | -0.107 | 0.054 |
| **Total CrCl (L/week/1.73 m**2) | -0.250 | ＜0.001 |
| **RRF (ml/min)** | -0.333 | ＜0.001 |
| **Urine output (ml/24 h)** | -0.358 | ＜0.001 |
| **UF (ml/24 h)** | 0.361 | ＜0.001 |

Angpt-2/Angpt-1, angiopoietin-2/angiopoietin-1; r, correlation coefficient; LDL, lower-density lipoprotein; Log_10_ hs-CRP, the logarithm of high sensitivity C-reactive protein to the base 10; Kt/V urea, urea clearance index; CrCl, creatinine clearance; RRF, residual renal function; UF, ultrafiltration.

**Supplementary Table 2** Univariate Cox analysis for cardiovascular and all-cause mortality.

| **Variables** | **cardiovascular mortality** | | **all-cause mortality** | |
| --- | --- | --- | --- | --- |
|  | **HR (95% CI)** | **P-value** | **HR (95% CI)** | **P-value** |
| **Gender (Male)** | 1.345 (0.714-2.534) | 0.358 | 1.048 (0.744-1.476) | 0.788 |
| **Age (years)** | 1.077 (1.047-1.107) | ＜0.001 | 1.065 (1.049-1.081) | ＜0.001 |
| **Cardiovascular disease** | 9.104 (4.296-19.293) | ＜0.001 | 4.141 (2.900-5.915) | ＜0.001 |
| **Diabetes mellitus** | 4.039 (2.155-7.573) | ＜0.001 | 2.642 (1.842-3.737) | ＜0.001 |
| **Hemoglobin (g/L)** | 0.983 (0.965-1.002) | 0.077 | 0.990 (0.980-1.001) | 0.064 |
| **Serum albumin (g/L)** | 0.968 (0.903-1.037) | 0.352 | 0.973 (0.937-1.011) | 0.164 |
| **LDL cholesterol** | 1.029 (0.843-1.257) | 0.778 | 1.010 (0.897-1.137) | 0.874 |
| **Log_10_hs-CRP** | 1.997 (1.150-3.466) | 0.014 | 2.047 (1.515-2.767) | ＜0.001 |
| **RRF (ml/min)** | 1.033 (0.908-1.176) | 0.621 | 0.942 (0.869-1.021) | 0.148 |
| **Angpt-2/Angpt-1 ratio** | 2.204 (1.149-4.227) | 0.017 | 1.605 (1.135-2.268) | 0.007 |

HR, hazard ratio; 95%CI, 95% confidence interval; LDL, lower-density lipoprotein; Log_10_hs-CRP, the logarithm of high sensitivity C-reactive protein to the base 10; RRF, residual renal function; Angpt-2/Angpt-1, angiopoietin-2/angiopoietin-1.
