# Supplementary figures and images for: Serum angiopoietin-2/angiopoietin-1 ratio is associated with cardiovascular and all-cause mortality in peritoneal dialysis patients: a prospective cohort study
Source: Ren Fail. 2024 Jul 31;46(2):2380037. doi: 10.1080/0886022X.2024.2380037 (PMC11293270; doi:10.1080/0886022X.2024.2380037)

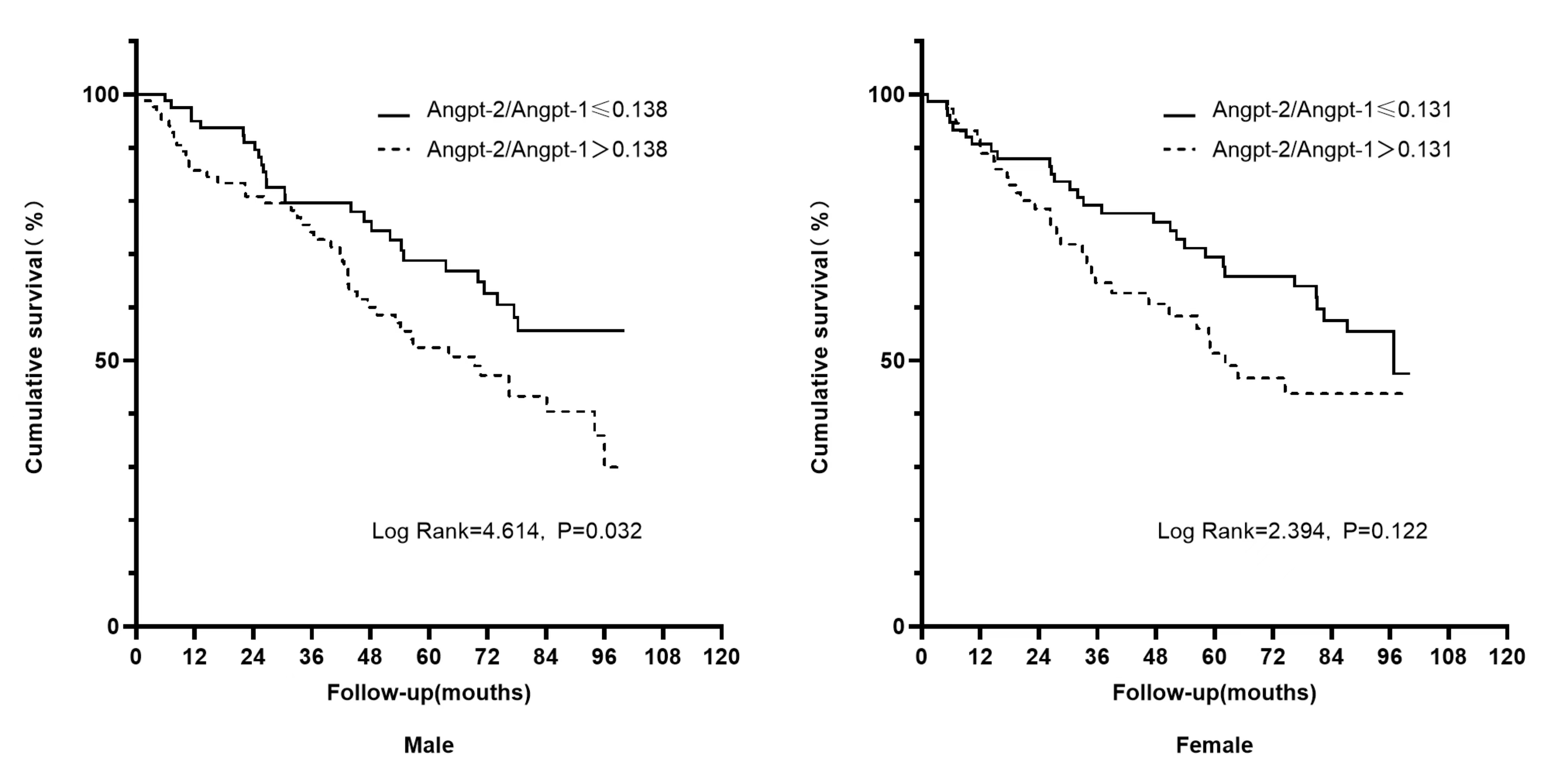

Supplement: Figure 2.jpg [file IRNF_A_2380037_SM7002.jpg]

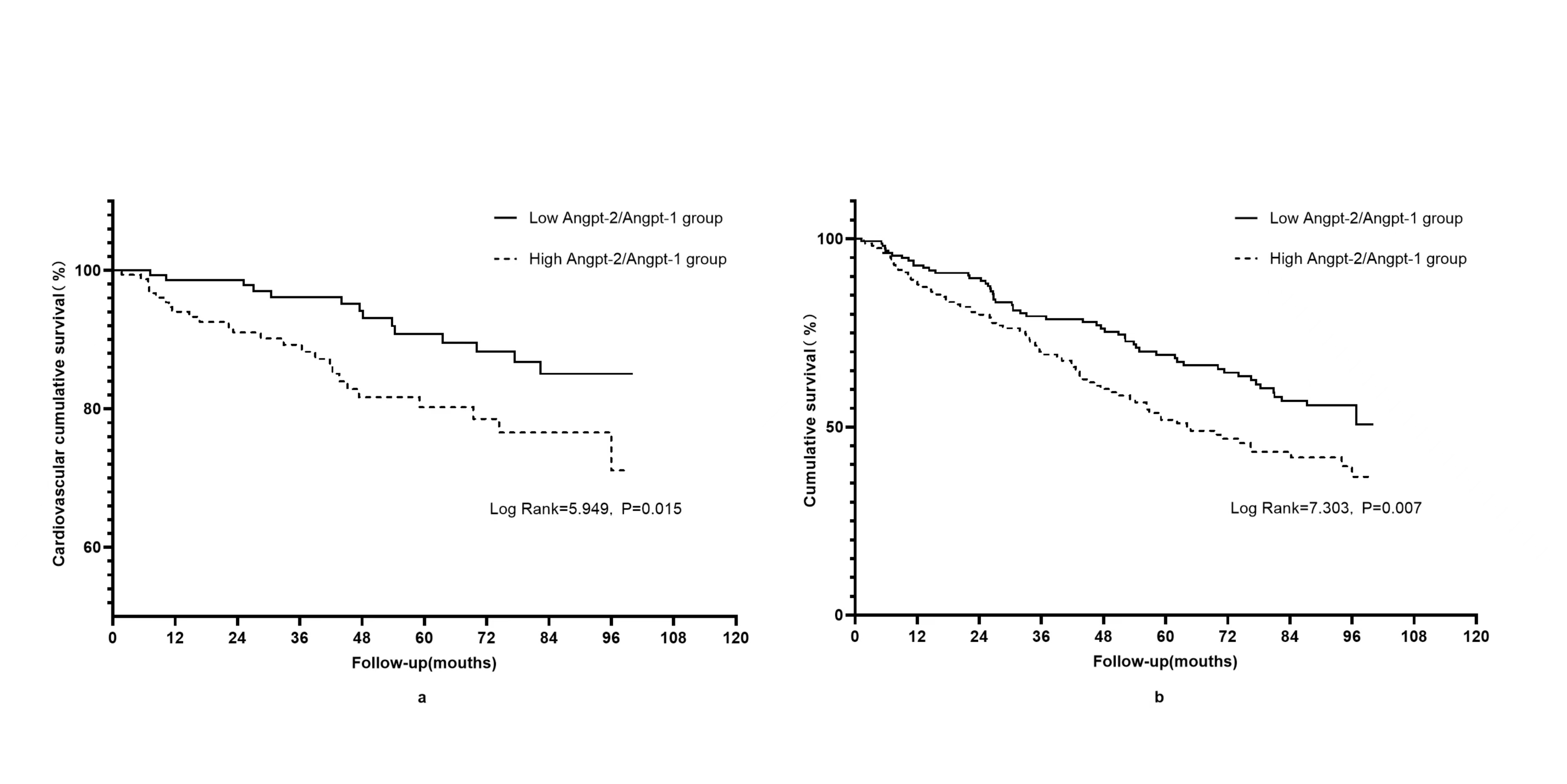

Supplement: Figure 1.jpg [file IRNF_A_2380037_SM7000.jpg]
