# Supplementary material for: Serum angiopoietin-2/angiopoietin-1 ratio is associated with cardiovascular and all-cause mortality in peritoneal dialysis patients: a prospective cohort study
Source: Ren Fail. 2024 Jul 31;46(2):2380037. doi: 10.1080/0886022X.2024.2380037 (PMC11293270; doi:10.1080/0886022X.2024.2380037)
Supplement: Supplementary table.docx [file IRNF_A_2380037_SM7001.docx]

**Supplementary table 1** Multivariate Cox regression models for all-cause mortality of male and female participants.

| **Variables** | **Male (n=168)** | | **Female (n=157)** | | | |
| --- | --- | --- | --- | --- | --- | --- |
|  | **HR (95% CI)** | **P-value** | | **HR (95% CI)** | **P-value** |  |
| **Age (years)** | 1.051(1.027-1.075) | ＜0.001 | | 1.048(1.021-1.075) | ＜0.001 |  |
| **Cardiovascular disease** | 3.965(2.258-6.963) | ＜0.001 | | 2.472(1.364-4.478) | 0.003 |  |
| **Diabetes mellitus** | 1.252(0.736-2.128) | 0.407 | | 1.465(0.794-2.704) | 0.221 |  |
| **Hemoglobin (g/L)** | 0.994(0.979-1.008) | 0.399 | | 0.997(0.982-1.012) | 0.667 |  |
| **Serum albumin (g/L)** | 0.983(0.929-1.039) | 0.542 | | 0.975(0.915-1.039) | 0.436 |  |
| **Log_10_hs-CRP** | 1.489(0.914-2.425) | 0.110 | | 1.084(0.648-1.813) | 0.760 |  |
| **RRF (ml/min)** | 0.903(0.797-1.023) | 0.111 | | 0.927(0.795-1.080) | 0.329 |  |
| **Angpt-2/Angpt-1 ratio** | 1.896(1.142-3.148) | 0.013 | | 1.223(0.708-2.113) | 0.470 |  |

HR, hazard ratio; 95%CI, 95% confidence interval; Log_10_hs-CRP, the logarithm of high sensitivity C-reactive protein to the base 10; RRF, residual renal function; Angpt-2/Angpt-1, angiopoietin-2/angiopoietin-1.
